# Combined association of Presenilin-1 and Apolipoprotein E polymorphisms with maternal meiosis II error in Down syndrome births

**DOI:** 10.1590/1678-4685-GMB-2016-0138

**Published:** 2017-07-31

**Authors:** Pranami Bhaumik, Priyanka Ghosh, Sujay Ghosh, Eleanor Feingold, Umut Ozbek, Biswanath Sarkar, Subrata Kumar Dey

**Affiliations:** 1 Department of Biotechnology, School of Biotechnology and Biological Sciences. Maulana Abul Kalam Azad University of Technology, West Bengal, India; 2 Department of Zoology, University of Calcutta, Ballygunge Science college campus, Kolkata, West Bengal, India; 3 Department of Human Genetics, Graduate School of Public Health, University of Pittsburgh, PA, USA; 4 Department of Biostatistics, Graduate School of Public Health, University of Pittsburgh, Pittsburgh, PA, USA; 5 DNA Laboratory, Anthropological Survey of India, Kolkata, India

**Keywords:** Chromosome, genetic polymorphism, karyotype, meiosis, microsatellite markers

## Abstract

Alzheimer's disease and Down syndrome often exhibit close association and predictively share common genetic risk-factors. Presenilin-1 (*PSEN-1*) and Apolipoprotein E (*APOE*) genes are associated with early and late onset of Alzheimer's disease, respectively. Presenilin −1 is involved in faithful chromosomal segregation. A higher frequency of the *APOE* ε4 allele has been reported among young mothers giving birth to Down syndrome children. In this study, 170 Down syndrome patients, grouped according to maternal meiotic stage of nondisjunction and maternal age at conception, and their parents were genotyped for *PSEN-1* intron-8 and *APOE* polymorphisms. The control group consisted of 186 mothers of karyotypically normal children. The frequencies of the *PSEN-1* T allele and TT genotype, in the presence of the *APOE* ε4 allele, were significantly higher among young mothers (< 35 years) with meiosis II nondisjunction than in young control mothers (96.43% *vs.* 65.91% *P* = 0.0002 and 92.86% *vs.* 45.45% *P* < 0.0001 respectively) but not among mothers with meiosis I nondisjunction. We infer that the co-occurrence of the *PSEN-1* T allele and the *APOE* ε4 allele associatively increases the risk of meiotic segregation error II among young women.

## Introduction

Alzheimer's disease (AD), a progressive neurodegenerative disorder of old age, and Down syndrome (DS), an intellectual disability due to trisomy of chromosome 21, show co-occurrence. Brain imaging and autopsy studies revealed that Alzheimer's-like neuropathological changes, such as beta amyloid plaques and neurofibrillary tangles were common in DS patients at their forties (Olson and Shaw, 1969; Glenner and Wang, 1984; Mann and Esiri, 1989; Cork, 1990; Yoshimura *et al.*, 1990). The common molecular mechanisms bridging the two disorders include chromosomal missegregation (Potter, 1991; 2008), overproduction of amyloid precursor protein (Rumble *et al.*, 1989), oxidative stress and mitochondrial dysfunction (Pagano and Castello, 2012), nuclear factor of activated T cells (NFAT) and tau phosphorylation pathways (Jung *et al.*, 2011; Perluigi *et al.*, 2014), endocytic pathway abnormality (Cataldo *et al.*, 2000), mutation in amyloid precursor protein gene (*APP*) (van Leeuwen *et al.*, 1998), presence of Apolipoprotein E epsilon 4 (*APOE* ε4) allele (Del Bo *et al.*, 1997). Familial association of AD and DS has been reported (Yatham *et al.*, 1988; Schupf *et al.*, 2001). Interactions among environmental agents, advancing age (Tanzi and Bertram, 2001; Grant *et al.*, 2002) and a certain genetic polymorphisms (Bertram and Tanzi, 2005) account for 95% of sporadic late-onset AD, while only 5% AD are of early-onset type and due to mutations in *APP* (Goate *et al.*, 1991), presenilin-1 (*PSEN-1*) (Sherrington *et al.*, 1995) and presenilin-2 (*PSEN-2*) (Levy-Lahad *et al.*, 1995; Rogaev *et al.*, 1995) genes on chromosome 21, 14 and 1, respectively. The *PSEN-1* gene encodes a protein component of the gamma-secretase complex involved in the processing of the amyloid precursor protein (APP) (Karran *et al.*, 1998). Presenilin-1 protein is engaged in many cardinal mechanisms of several molecular pathways (Duff *et al.*, 1996; Alberici *et al.*, 1999; Woo *et al.*, 2009; Ho and Shen, 2011; Trushina *et al.*, 2012), which when impaired lead to the manifestation of AD. This protein also localizes to centromeres, the nuclear envelope of dividing cells, kinetochores at interphase, and is involved in faithful chromosomal segregation (Li *et al.*, 1997). Mutations in *PSEN-1* lead to chromosomal instability and trisomy 21 mosaicism in AD patients (Geller and Potter, 1999). Another well-documented molecular marker for both the early-onset (Corder *et al.*, 1993) and sporadic (Brouwers *et al.*, 2008) AD is a polymorphism in the Apolipoprotein E (*APOE*) gene on chromosome 19. Association of the *APOE* ε4 allele with AD has been demonstrated in ethnically different populations (Lehtimaki *et al.*, 1995; Shimada *et al.*, 1997; Tang *et al.*, 1998; Panza *et al.*, 1999; de-Andrade *et al.*, 2000; Kim *et al.*, 2001; Korovaitseva *et al.*, 2001; Chen *et al.*, 2003). On the other hand, DS is the most common aneuploidy in live born humans. The predominant cause of DS is the presence of a supernumerary chromosome 21, owing to nondisjunction in maternal gametogenesis in the overwhelming majority of cases (Sherman *et al.*, 2007; Allen *et al.*, 2009; Ghosh *et al.*, 2010). Advanced maternal age (Hassold and Chiu, 1985; Allen *et al.*, 2009) and an altered pattern of recombination (Warren *et al.*, 1987; Sherman *et al.*, 1991; Oliver *et al.*, 2008) have been identified as two major risk factors for maternal meiotic errors. Avramopoulos *et al.* (1996) found a higher of the *APOE* ε4 allele in young mothers having DS children due to chromosomal nondisjunction in the second meiotic division (meiosis II or MII) of oocytes. The association of *PSEN-1* intron 8 polymorphism and late-onset AD in North American European descendants was first reported by Wragg *et al.* (1996) and later supported in many studies (Higuchi *et al.*, 1996; Isoe *et al.*, 1996; Kehoe *et al.*, 1996; Brookes *et al.*, 1997; Ezquerra *et al.*, 1997; Nishiwaki *et al.*, 1997; Tilley *et al.*, 1999); arguments against this association were also produced (Pérez-Tur *et al.*, 1996; Scott *et al.*, 1996; Cai *et al.*, 1997; Lendon *et al.*, 1997; Singleton *et al.*, 1997; Sorbi *et al.*, 1997; Tysoe *et al.*, 1997; Jiang *et al.*, 1999; Bagli *et al.*, 1999; Rodriguez *et al.*, 2000; Chandak *et al.*, 2002; Rassas *et al.*, 2013). The study of a DS sample from Denmark revealed the association of the T allele of the *PSEN-1* intronic polymorphism (rs165932) with maternal MII nondisjunction, and thus pointed to a putative role of this polymorphic allele in chromosomal segregation (Petersen *et al.*, 2000). The aim of the present study was to investigate the possibility of a collaborative effect of *PSEN-1* and *APOE* polymorphisms on DS birth in the Indian subcontinent.

## Subjects and Methods

### Subjects

This study included 178 unrelated Bengali individuals with free trisomy 21 and their parents. We recruited 186 women that gave birth to karyotypically normal children as the control group. All subjects were randomly referred from different Medical Colleges and Hospitals of Kolkata and neighbouring areas. The study was approved by the ethical committee of the Maulana Abul Kalam Azad University of Technology. Peripheral blood was collected from the DS children and their parents, as well as from control mothers and their children after taking informed consent.

### Cytogenetic analysis

Classical karyotyping was performed to select only free trisomy 21 DS cases. At least 30 metaphases were analysed from each DS sample to exclude mosaicism.

### Determination of parental origin of extra chromosome 21

Genomic DNA was isolated from blood using a QIAamp DNA Blood Midi Kit (Qiagen). Ten highly polymorphic STR markers, mapped from the pericentromeric region to the telomeric region of the long arm of chromosome 21were selected to determine the maternal or paternal origin of the extra chromosome 21: D21S1432 – D21S11 – D21S1437 – D21S1270 –D21S167 – D21S1412 – D21S2055 – D21S1260 – D21S1411 – D21S1446. For determining the stage of meiotic nondisjunction, *i.e.* MI or MII errors, four additional pericentromeric markers were genotyped: D21S369, D21S215, D21S258 and D21S120. The maternal MI error was inferred, when maternal heterozygosity for these markers was retained in the DS child. If maternal heterozygosity was reduced to homozygosity in the DS child, maternal MII error was considered.

### Detection of *APOE* and *PSEN-1* polymorphisms

Polymorphisms in *APOE* gene (*rs*429358 and rs7412) and *PSEN-1* intron 8 (rs165932) were investigated by Restriction Fragment Length Polymorphism (RFLP), and direct DNA sequencing in an ABI PRISM 3700 DNA Analyzer platform (Applied Biosystems), after PCR amplification, using oligonucleotide primers previously described by Hixson and Vernier (1990) and Sherrington *et al.* (1995), respectively. Restriction fragment length polymorphism (RFLP) genotyping of *APOE* and *PSEN-1* was done, as described by Hixson and Vernier (1990) and Wragg *et al.* (1996) respectively.

### Statistical analysis

Maternal age was considered as predictor variable in all statistical analyses. For age analyses, both case and control mothers were stratified into young (< 35 years) and old (> 35 years) groups. Chi-squared tests were performed to compare genotypic and allelic frequencies between case and control mothers, as well as between MI and MII nondisjunction groups, as distinct molecular mechanisms are supposed to be responsible for these errors.

Considered the high number of statistical tests used to compare the many partitions and combinations we created from our original groups of control and DS mothers, the alpha critical level obtained by a simple Bonferroni correction was set at 0.0005. Since the partitions and rearrangements of the total samples of control and DS mothers were somewhat correlated, we reset this value at the less stringent level alpha = 0.001.

## Results

STR genotyping revealed that out of the 178 DS trisomies only eight had a paternal meiotic origin, and 170 were the result of maternal nondisjunction. MI nondisjunction was demonstrated in 106 cases (53 young mothers and 53 old mothers), and MII nondisjunction in 64 cases (33 young mothers and 31 old mothers). According to the presence of the *APOE* ε4 allele, stage of nondisjunction and age at conception, the 170 case- mothers were stratified into eight groups : (a) ε4 positive, - MI, - Young, n = 16; (b) ε4 positive, - MI, - Old, n = 13; (c) ε4 positive, - MII, - Young, n = 14; (d) ε4 positive, - MII, - Old, n = 8; (e) ε4 negative, - MI, - Young, n = 37; (f) ε4 negative, - MI, - Old, n = 40; (g) ε4 negative, - MII, - Young, n = 19; (h) ε4 negative, - MII, - Old, n = 23. The control mothers of karyotypically normal children were also categorised as: (a) ε4 positive, - Young, n = 22; (b) ε4 positive, - Old, n = 20; (c) ε4 negative, - Young, n = 71; (d) ε4 negative, - Old, n = 73. The distribution of *PSEN-1* alleles and genotypes in each group of case and control mothers are presented in Supplementary Tables S1 and S2, respectively. All groups were in Hardy-Weinberg equilibrium.

### *PSEN-1* polymorphism and maternal age

Stratified analyses for meiotic outcome groups revealed that the TT genotype was significantly more frequent in the group of young mothers with MII nondisjunction compared to young control mothers. (*P* = 0.0007; Table 1).

**Table 1:** Comparison of *PSEN-1* TT Genotypic and T allelic frequencies among different groups of mothers of DS children and control mothers of karyotypically normal children.

Comparisons	TT genotypic frequency		*P* value of Chi–squared test	T allelic frequency		*P* value of Chi–squared test
	Case	Control		Case	Control	
Case mothers (N = 170) *vs.*	55.29%	48.92%	0.36	72.35%	68.55%	0.65
Control mothers (N = 186)						
MI case mothers (N = 106) *vs.*	49.06%	48.92%	0.98	68.39%	68.55%	0.98
Control mothers (N = 186)						
MII case mothers (N = 64) *vs.*	65.63%	48.92%	0.02	78.91%	68.55%	0.21
Control mothers (N = 186)						
MII case mothers (N = 64) *vs.*	65.63%	49.06%	0.02	78.91%	68.39%	0.20
MI case mothers (N = 106)						
Young case mothers (N = 86) *vs.*	55.81%	44.09%	0.08	73.26%	63.98%	0.25
Young control mothers (N = 93)						
Old case mothers (N = 84) *vs.*	54.76%	53.76%	0.89	71.43%	73.12%	0.84
Old control mothers (N = 93)						
MI - young case mothers (N = 53) *vs.*	49.06%	44.09%	0.45	68.87%	63.98%	0.54
Young control mothers (N = 93)						
MI - old case mothers (N = 53) *vs.*	49.06%	53.76%	0.52	67.92%	73.12%	0.54
Old control mothers (N = 93)						
MII - young case mothers (N = 33) *vs.* Young control mothers (N = 93)	66.67%	44.09%	0.0007	80.3%	63.98%	0.04
MII - old case mothers (N = 31) *vs.*	64.52%	53.76%	0.14	77.42%	73.12%	0.62
Old control mothers (N = 93)						
*APOE* ε4 positive - young case mothers (N = 30) *vs. APOE* ε4 positive - young control mothers (N = 22)	66.67%	45.45%	0.002	80%	65.91%	0.08
*APOE* ε4 positive - old case mothers	52.38%	55%	0.72	69.05%	72.5%	0.69
(N = 21) *vs. APOE* ε4 positive - old control mothers (N = 20)						
*APOE* ε4 negative - young case mothers (N = 56) *vs. APOE* ε4 negative - young control mothers (N = 71)	50%	43.66%	0.34	69.64%	63.38%	0.43
*APOE* ε4 negative - old case mothers	55.56%	53.42%	0.77	72.22%	73.29%	0.90
(N = 63) *vs. APOE* ε4 negative - old control mothers (N = 73)						
*APOE* ε4 positive - MI - young case mothers (N = 16) *vs. APOE* ε4 positive - young control mothers (N = 22)	43.75%	45.45%	0.80	65.62%	65.91%	0.97
*APOE* ε4 positive - MI - old case mothers (N = 13) *vs. APOE* ε4 positive - old control mothers (N = 20)	46.15%	55%	0.23	65.38%	72.5%	0.40
*APOE* ε4 negative - MI - young case mothers (N = 37) *vs. APOE* ε4 negative - young control mothers (N = 71)	51.35%	43.66%	0.24	70.27%	63.38%	0.39
*APOE* ε4 negative - MI - old case mothers (N = 40) *vs. APOE* ε4 negative - old control mothers (N = 73)	50%	53.42%	0.64	68.75%	73.29%	0.59
*APOE* ε4 positive - MII - young case mothers (N = 14) *vs. APOE* ε4 positive - young control mothers (N = 22)	92.86%	45.45%	<0.0001	96.43%	65.91%	0.0002
*APOE* ε4 positive - MII - old case mothers (N = 8) *vs. APOE* ε4 positive - old control mothers (N = 20)	62.5%	55%	0.31	75%	72.5%	0.77
*APOE* ε4 negative - MII - young case mothers (N = 19) *vs. APOE* ε4 negative - young control mothers (N = 71)	47.37%	43.66%	0.57	68.42%	63.38%	0.53
*APOE* ε4 negative - MII - old case mothers (N = 23) *vs. APOE* ε4 negative - old control mothers (N = 73)	65.22%	53.42%	0.11	78.26%	73.29%	0.56
*APOE* ε4 positive - MII case mothers	81.82%	44.83%	<0.0001	88.64%	65.52%	0.004
(N = 22) *vs. APOE* ε4 positive - MI case mothers (N = 29)						

Young mothers, < 35 yrs of age; Old mothers, < 35 yrs of age MI, nondisjunction at meiotic division I; MII, nondisjunction at meiotic division II

### *APOE* ε4 allele and nondisjunction

The detailed genotypes and alleles of *APOE* gene polymorphism in DS mothers and controls, according to age and meiotic nondisjunction stage are given in the Supplementary Table S3.

In young case mothers, the presence of ε4/- genotypes (i.e. ε4/ε4, ε3/ε4 or ε2/ε4) increased the risk for DS 1.73 times (Table 2). Both the allelic (ε4) and genotypic (ε4/ε4 + ε3/ε4 + ε2/ε4) frequencies were significantly increased in the MII nondisjunction young group when compared with young controls and with MI nondisjunction old group (*P* < 0.001, for genotypic and allelic frequencies). In the group of MII nondisjunction young mothers, the risk of nondisjunction was increased 2.48 times in the presence of the ε4 allele when compared with the group of MI nondisjunction old mothers (OR = 2.48, 95% CI = 1.11 – 5.53; Table 2) and 2.23 times when compared with young control mothers (OR = 2.23, 95% CI = 1.12 – 4.47; Table 2).

**Table 2:** Comparative analysis of *APOE* ε4/- genotypic and ε4 allelic frequencies in mothers of DS children and control mothers of karyotypically normal children.

	*APOE* ε4 positive genotypic frequency (ε4/ ε4 + ε3/ ε4 + ε2/ ε4)				*APOE* ε4 allelic frequency			
Comparisons	Chi square	*P* value of Chi - squared test	OR	95 % CI	Chi square	*P* value of Chi - squared test	OR	95 % CI
Case mothers (N= 170) vs. control mothers(N= 186)	2.44	0.12	1.47	0.91 -2.36	1.8	0.18	1.46	0.96 - 2.23
Young case mothers (N= 86) vs. Young control mothers (N= 93)	5.33	0.02	1.73	0.90 – 3.32	2.98	0.08	1.59	0.90 - 2.79
Old case mothers (N= 84) vs. Old control mothers (N= 93)	0.57	0.45	1.22	0.60 - 2.45	0.8	0.37	1.32	0.69 - 2.50
MI - young case mothers (N= 53) vs. Young control mothers (N= 93)	1.8	0.18	1.4	0.65 - 2.97	0.5	0.48	1.23	0.63 - 2.40
MI - old case mothers (N= 53) vs. Old control mothers (N= 93)	0.43	0.51	1.19	0.53 - 2.63	0.21	0.64	1.16	0.55 - 2.44
MII - young case mothers (N= 33) vs. Young control mothers (N= 93)	14.89	0.0001	2.38	1.03 - 5.51	11.29	0.0008	2.23	1.12 – 4.47
MII - old case mothers (N= 31) vs. Old control mothers (N= 93)	0.86	0.35	1.27	0.49 - 3.26	2.69	0.1	1.6	0.70 - 3.63
MII - Young case mothers (N= 33) vs. MI - old case mothers (N= 53)	13.06	0.0003	2.27	0.89 - 5.76	14.85	0.0001	2.48	1.11 - 5.53

Young mothers, < 35 yrs of age; Old mothers, > 35 yrs of age MI, nondisjunction at meiotic division I; MII, nondisjunction at meiotic division II

### Combined effect of the *PSEN-1* T allele and the *APOE* ε4 allele and maternal aging on non disjunction

We found a significant increase in both TT genotypic and T allelic frequencies in *APOE* ε4 positive, - MII nondisjunction,- young case mothers upon comparison with *APOE* ε4 positive, - young control mothers (*P* < 0.00001 and 0.0002, respectively; Table 1).

These results suggest that the *PSEN-1* T allele and the *APOE* ε4 allele may collaboratively increase the risk of MII nondisjunction among young mothers.

## Discussion

The aim of the present work was to explore the notion that the etiology of DS birth and AD is somehow related at the molecular level. The result of our analyses suggested that polymorphisms of *PSEN-1* might explain the co-occurrence of DS and AD in one same family.

The result of our case control study showed that the ‘T allele’ of *PSEN-1* intronic polymorphism (rs165932) was associated with MII nondisjunction, but not with MI nondisjunction. It is not clear at this point how this polymorphism impacts the chromosome segregation, but two hypotheses have been put forward to explain its molecular role. According to the first hypothesis, the *PSEN-1* intron 8 T allele may be in linkage disequilibrium with a coding segment in the gene itself or in other gene(s) (Hutton and Hardy, 1997); and the second hypothesis postulates that this polymorphic site may affect the pre-mRNA splicing and give rise to a different isoform of the protein, which may affect chromosome segregation (Meshorer and Soreq, 2002). Abnormality in cell cycle regulation is apparent in both familial and sporadic AD cases (Potter, 1991, 2005, 2008; Arendt *et al.*, 1996; Geller and Potter, 1999; Yang *et al.*, 2001, 2006; Nagy, 2005; Yang and Herrup, 2007; Varvel *et al.*, 2008).

The significant increase in T allelic and TT genotypic frequencies in ε4 positive young mothers with MII nondisjunction would imply a collaborative effect of both alleles in increasing the risk of MII nondisjunction at young age. Avramopoulos *et al.* (1996) found higher *APOE* ε4 allele frequencies in young mothers giving birth to DS child due to meiotic II nondisjunction error. This would be explained by compromised microcirculation due to the high plasma cholesterol deposition in *APOE* ε4 allele carriers causing atherosclerosis in microvasculature surrounding ovarian follicles. This would imply reduced blood flow and oxygen supply, and increased anaerobic products such as lactic acid accumulation in the follicular cell and as a consequence the size of the spindle, could become reduced due to high pH inside the follicle, resulting in nondisjunction (Gaulden, 1992). Another explanation is that isoform-specific binding of ApoE to microtubule-associated protein would affect microtubule stability and function and, thus, hamper meiotic chromosomal segregation (Strittmatter *et al.*, 1993, 1994; Hansen *et al.*, 1998). Support to this prediction has been provided by Nagy *et al.* (2000), who showed that trisomy 13 and trisomy 21 conceptuses have a higher *APOE* ε4 allele frequency.

A recent study has shown that *APOE* regulates telomere dynamics, and the females who carry *APOE* ε4 allele experience a six-times higher rate of telomere shortening than non-carriers (Jacobs *et al.*, 2013). Greater erosion of telomere length in Alzheimer's patients with *APOE* ε4 allele is also evident (Takata *et al.*, 2012). Interestingly, the study of Ghosh *et al.* (2010) revealed higher degree of telomere loss in mothers of DS patients resulting from MII nondisjunction than in MI nondisjunction cases and controls. But it is difficult at this point to explain how these data fit together.

Taking all the above into account, we may conclude that the T allele and TT genotype of *PSEN-1* polymorphism is associated with MII nondisjunction in younger women giving birth to DS children. Petersen *et al.* (2000) reported similar findings in Denmark. This result is somewhat interesting as we (Ghosh *et al.*, 2009) and others (Oliver *et al.*, 2008) have found that MII nondisjunction is frequent among older mothers, and represents a maternal age dependent phenomenon. The present set of results suggests that MII nondisjunction can be a maternal age independent phenomenon, when mothers carry the *APOE* ε4 and *PSEN-1* T alleles. The gradual increase in the association of the three factors - *PSEN-1* T allele, *APOE* ε4 allele and young age with MII nondisjunction but not with MI nondisjunction, suggests that these two errors are mutually exclusive and involve different molecular mechanisms. Considering the findings of previous studies (Oliver *et al.*, 2008; Ghosh *et al.*, 2009) and the present data together, we could infer predictively that *APOE* ε4 allele and *PSEN-1* rs165932 T allele create a microenvironment in the younger oocyte, which mimics the subcellular condition of chronologically older ovum and causes MII nondisjunction, a possibility warranting confirmation through elaborate molecular study. Nevertheless, our study provides the first independent confirmation of *PSEN-1* as the prospective molecular candidate that relates AD with DS. The association of the T allele of *PSEN-1* intronic polymorphism (rs165932) and the *APOE* ε4 allele would be the collaborative risk factor for both AD and DS, reciprocally exacerbating the risk of MII nondisjunction. Moreover, for the very first time we have clearly demonstrated that the distribution of risk alleles is statistically similar among controls and MI nondisjunction groups. These results being in accordance with those of Peterson *et al.* (2000) suggest that the molecular risk factor underlying the association of AD and DS is independent of ethnicity. Our findings represent a step towards the understanding of the genetic basis of DS birth and AD occurance within one same family.

## Supplementary material

The following online material is available for this article:

Table S1 - *PSEN-1* genotypic and allelic frequencies in mothers of DS children.

Table S2 - *PSEN-1* genotypic and allelic frequencies in control mothers.

Table S3 - *APOE* genotypic and allelic frequencies in mothers of DS children and control mothers.
