# Supplementary material for: Combined association of Presenilin-1 and Apolipoprotein E polymorphisms with maternal meiosis II error in Down syndrome births
Source: Genet Mol Biol. 2017 Jul 31;40(3):577–85. doi: 10.1590/1678-4685-GMB-2016-0138 (PMC5596362; doi:10.1590/1678-4685-GMB-2016-0138)
Supplement: Supplementary file 1 [file 1415-4757-gmb-1678-4685-GMB-2016-0138-Suppl01.pdf]

Supplementary Material to "Combined association of Presenilin-1 and Apolipoprotein E polymorphisms with maternal meiosis II error in Down syndrome births"

Table S1 - *PSEN-1* genotypic and allelic frequencies in mothers of DS children.

Subjects		Genotypes TT	TG	GG	Alleles T	G
Total mothers (N = 170)		0.5529	0.3412	0.1059	0.7235	0.2764
Young mothers (N = 86)		0.5581	0.3488	0.093	0.7326	0.2674
Old mothers (N = 84)		0.5476	0.3333	0.119	0.7143	0.2857
APOE ε4 - positive mothers (N = 51)		0.6078	0.2941	0.098	0.7549	0.2451
APOE ε4 - negative mothers (N = 119)		0.5294	0.3613	0.1092	0.7101	0.2899
APOE ε4 - positive young mothers (N = 30)		0.6667	0.2667	0.0667	0.8	0.2
APOE ε4 - positive old mothers (N = 21)		0.5238	0.3333	0.1429	0.6905	0.3095
APOE ε4 - negative young mothers (N = 56)		0.5	0.3929	0.1071	0.6964	0.3036
APOE ε4 - negative old mothers (N = 63)		0.5556	0.3333	0.1111	0.7222	0.2778
Mothers with M I nondisjunction (N = 106)		0.4906	0.3868	0.1226	0.6839	0.316
Young mothers with M I nondisjunction (N = 53)		0.4906	0.3962	0.1132	0.6887	0.3113
Old mothers with M I nondisjunction (N = 53)		0.4906	0.3774	0.1321	0.6792	0.3208
Mothers with M II nondisjunction (N = 64)		0.6563	0.2656	0.0781	0.7891	0.2109
Young mothers with M II nondisjunction (N = 33)		0.6667	0.2727	0.0606	0.803	0.1969
Old mothers with M II nondisjunction (N = 31)		0.6452	0.2581	0.0968	0.7742	0.2258
ε4 positive mothers N=51	M I nondisjunction N=29	0.4483	0.4138	0.1379	0.6552	0.3448
	MI nondisjunction – young mothers N=16	0.4375	0.4375	0.125	0.6562	0.3438
	MI nondisjunction – old mothers N=13	0.4615	0.3846	0.1538	0.6538	0.3462
	M II nondisjunction N=22	0.8182	0.1364	0.0455	0.8864	0.1136
	MII nondisjunction – young mothers N=14	0.9286	0.0714	0	0.9643	0.0357
	MII nondisjunction – old mothers N=8	0.625	0.25	0.125	0.75	0.25
ε4 negative mothers N= 119	M I nondisjunction N=77	0.5065	0.3766	0.1169	0.6948	0.3052
	MI nondisjunction – young mothers N=37	0.5135	0.3784	0.1081	0.7027	0.2973
	MI nondisjunction – old mothers N=40	0.5	0.375	0.125	0.6875	0.3125
	M II nondisjunction N=42	0.5714	0.3333	0.0952	0.7381	0.2619
	MII nondisjunction – young mothers N=19	0.4737	0.4211	0.1053	0.6842	0.3158

Subjects	Genotypes			Alleles	
	TT	TG	GG	T	G
MII nondisjunction – old mothers N= 23	0.6522	0.2609	0.0869	0.7826	0.2174

Young mothers, < 35 yrs of age; Old mothers, > 35 yrs of age
