# Supplementary material for: Combined association of Presenilin-1 and Apolipoprotein E polymorphisms with maternal meiosis II error in Down syndrome births
Source: Genet Mol Biol. 2017 Jul 31;40(3):577–85. doi: 10.1590/1678-4685-GMB-2016-0138 (PMC5596362; doi:10.1590/1678-4685-GMB-2016-0138)
Supplement: Supplementary file 2 [file 1415-4757-gmb-1678-4685-GMB-2016-0138-Suppl02.pdf]

Genetics and Molecular Biology, 40, 3 (2017)

Supplementary Material to "Combined association of Presenilin-1 and Apolipoprotein E polymorphisms with maternal meiosis II error in Down syndrome births"

Table S2 - *PSEN-1* genotypic and allelic frequencies in control mothers of karyotypically normal children.

**Subjects**	**Genotypes**			**Alleles**	
	**TT**	**TG**	**GG**	**T**	**G**
Control mothers (N = 186)	0.4892	0.3925	0.1183	0.6855	0.3145
Young mothers (N = 93)	0.4409	0.3978	0.1613	0.6398	0.3602
Old mothers (N = 93)	0.5376	0.3871	0.0753	0.7312	0.2688
APOE ε4 - positive mothers (N=42)	0.5	0.3809	0.119	0.6905	0.3095
APOE ε4 - positive young mothers N= 22	0.4545	0.4091	0.1364	0.6591	0.3409
APOE ε4 - positive old mothers N= 20	0.55	0.35	0.1	0.725	0.275
APOE ε4 - negative mothers (N=144)	0.4861	0.3958	0.1181	0.684	0.3159
APOE ε4 - negative young mothers N=71	0.4366	0.3944	0.169	0.6338	0.3662
APOE ε4 - negative old mothers N= 73	0.5342	0.3973	0.0685	0.7329	0.2671

Young mothers, < 35 yrs of age; Old mothers, > 35 yrs of age
