# Supplementary material for: Combined association of Presenilin-1 and Apolipoprotein E polymorphisms with maternal meiosis II error in Down syndrome births
Source: Genet Mol Biol. 2017 Jul 31;40(3):577–85. doi: 10.1590/1678-4685-GMB-2016-0138 (PMC5596362; doi:10.1590/1678-4685-GMB-2016-0138)
Supplement: Supplementary file 3 [file 1415-4757-gmb-1678-4685-GMB-2016-0138-Suppl03.pdf]

Supplementary Material to "Combined association of Presenilin-1 and Apolipoprotein E polymorphisms with maternal meiosis II error in Down syndrome births"

Table S3 - *APOE* genotypic and allelic frequencies in mothers of DS children and control mothers.

**Subjects**	***APOE* Genotypes**						***APOE* Alleles**		
	**ε2/ ε2**	**ε2/ ε3**	**ε3/ ε3**	**ε3/ ε4**	**ε4/ ε4**	**ε2/ ε4**	**ε2**	**ε3**	**ε4**
Mothers of DS children (N = 170)	0.0059	0.0529	0.6412	0.2353	0.0353	0.0294	0.0471	0.7853	0.1676
Control mothers (N = 186)	0.0054	0.0538	0.7151	0.1935	0.0161	0.0161	0.0403	0.8387	0.121
Young mothers of DS children (N = 86)	0.0116	0.0465	0.593	0.2558	0.0465	0.0465	0.0581	0.7442	0.1977
Young control mothers (N = 93)	0	0.043	0.7204	0.172	0.0323	0.0323	0.0376	0.828	0.1344
Old mothers of DS children (N = 84)	0	0.0595	0.6905	0.2143	0.0238	0.0119	0.0357	0.8274	0.1369
Old control mothers (N = 93)	0.0108	0.0645	0.7097	0.2151	0	0	0.0430	0.8495	0.1075
Mothers of DS children with M I nondisjunction (N = 106)	0.0094	0.066	0.6509	0.2453	0.0094	0.0189	0.0519	0.8066	0.1415
Young mothers of DS children with M I nondisjunction (N = 53)	0.0189	0.0755	0.6038	0.2642	0.0189	0.0189	0.066	0.7736	0.1604
Old mothers of DS children with M I nondisjunction (N = 53)	0	0.0566	0.6981	0.2264	0	0.0189	0.0377	0.8396	0.1226
Mothers of DS children	0	0.0313	0.625	0.2188	0.0781	0.0469	0.0391	0.75	0.2109

Subjects	*APOE* Genotypes						*APOE* Alleles		
	**ε2/ ε2**	**ε2/ ε3**	**ε3/ ε3**	**ε3/ ε4**	**ε4/ ε4**	**ε2/ ε4**	**ε2**	**ε3**	**ε4**
with M II nondisjunction (N = 64)									
Young mothers of DS children with M II nondisjunction (N = 33)	0	0	0.5758	0.2424	0.0909	0.0909	0.0454	0.6969	0.2576
Old mothers of DS children with M II nondisjunction (N = 31)	0	0.0645	0.6774	0.1935	0.0645	0	0.0323	0.8065	0.1613

Young mothers, < 35 yrs of age; Old mothers, > 35 yrs of age
